# Complete Genome Sequence of an Endemic Mycobacterium bovis Strain from Wood Bison in Wood Buffalo National Park, Canada

**DOI:** 10.1128/mra.00097-23

**Published:** 2023-04-17

**Authors:** Olga Andrievskaia, Todd Shury, Amalia Garceac, Dara Lloyd, Kristin Arnold, Marc-Olivier Duceppe

**Affiliations:** a Canadian Food Inspection Agency, Ottawa Laboratory Fallowfield, Ottawa, Ontario, Canada; b Parks Canada Agency, Saskatoon, Saskatchewan, Canada; Wellesley College

## Abstract

Mycobacterium bovis is the primary causative agent of bovine tuberculosis, a zoonotic infectious disease of concern for human health, livestock, and wildlife conservation. We report a complete genome sequence of an endemic Mycobacterium bovis strain affiliated with a wildlife reservoir of bovine tuberculosis found in wood bison in Wood Buffalo National Park, Canada.

## ANNOUNCEMENT

Mycobacterium bovis is the most common causative agent of bovine tuberculosis (bTb), an important zoonotic disease (1). While bTb is considered to have been eliminated from Canadian livestock (2), a population of free-ranging wood bison (Bison bison *athabascae*) in and around Wood Buffalo National Park (WBNP) acts as a wildlife bTb reservoir (3, 4). We report a complete genome sequence of an M. bovis strain that originated in WBNP to provide a reference for future epidemiological investigations and disease surveillance. Mycobacteriosis-compatible granulomatous tissue samples were collected at necropsy of a wood bison bull in WBNP (59.0436009, −112.402869) by Parks Canada Agency wildlife health staff in March 2022 and submitted for bacterial culture to the National Reference Laboratory for Bovine Tuberculosis, Canadian Food Inspection Agency. The samples were collected under Parks Canada research and collection permit number 45863. The tissue samples were processed for inoculation into several types of liquid and solid media and incubated at 37°C until typical mycobacterial colonies were observed and characterized according to standard microbiological methods (5). M. bovis strain 2022/0038 (OLF-AH-2022-TB-0122) was isolated from modified Middlebrook with malachite green (MMMG) medium and taxonomically identified using biochemical (5) and molecular (6) tests, including niacin/nitrate biochemical testing, species-specific PCRs, and partial 16S rRNA gene and *rpoB* gene sequencing. Genomic DNA (gDNA) was purified from an MMMG subculture using the MasterPure Gram-positive DNA purification kit (Lucigen, USA). An Illumina sequencing library was prepared using the Nextera XT DNA library preparation kit (Illumina Inc., USA), and 2,643,748 paired-end 250-bp reads were generated on the Illumina MiSeq platform. For Nanopore sequencing, gDNA samples were additionally treated with the short-read eliminator XS kit (Circulomics, USA). The Nanopore library was prepared using the ligation sequencing gDNA kit (SQK-LSK109; Oxford Nanopore Technologies [ONT], UK) without shearing and sequenced on a Flongle FLO-FLG001 flow cell using the MinION Mk1B device (ONT), generating 71,783 reads with an *N*_50_ value of 5,978 bp. Base calling of the Nanopore reads was performed using Guppy v6.3.7 in super-accuracy mode. The reads were trimmed using Porechop v0.2.4 and filtered using Filtlong v0.2.1 (7). Long-read assembly was performed with Trycycler v0.5.3 (8) using Flye v2.9 (9), NECAT v0.0.1 (10), Canu v2.2 (11), and Shasta v0.8.0 (12). The reads were split into six subsamples. Trycycler-generated circularized consensus sequences were corrected using Medaka v1.4.4 and polished with the Illumina MiSeq reads using a combination of NextPolish v1.4.0 (13), ntEdit v1.3.5 (14), and Polypolish v0.5.0 (15) after trimming/filtering using fastp v0.23.2 (16). The sequencing coverage depth was determined using Minimap2 v2.17 (17) and SAMtools v1.15.1 (18) for the Nanopore reads (44×) and BWA v0.7.17 and SAMtools for the Illumina reads (94×). Default parameters of all bioinformatics software were used unless otherwise specified (https://github.com/OLF-Bioinformatics/nanopore/blob/master/run_trycycler.sh; https://github.com/OLF-Bioinformatics/nanopore/blob/master/short_read_polish.sh). Gene prediction and annotation were performed using the NCBI Prokaryotic Genome Annotation Pipeline v6.4 (19). The complete M. bovis 2022/0038 genome contains a single 4,342,292-bp chromosome, with a 65.6% G+C content, 4,061 predicted genes, 45 tRNAs, and 2 clustered regularly interspaced short palindromic repeats (CRISPRs). Whole-genome comparison of the strain 2022/0038 with the M. bovis reference strain AF2122/97 (20) using FastANI v1.33 (21) generated an average nucleotide identity of 99.98%.

### Data availability.

The Illumina and Nanopore sequencing reads are available in the NCBI Sequence Read Archive under accession numbers SRR23325069 and SRR23325070, respectively. The complete genome sequence of WBNP M. bovis strain 2022/0038 (OLF-AH-2022-TB-0122) has been deposited at GenBank under accession number CP117298.
